# Supplementary material for: Epidermal growth factor-like domain 7 drives brain lymphatic endothelial cell development through integrin αvβ3
Source: Nat Commun. 2024 Jul 16;15:5986. doi: 10.1038/s41467-024-50389-8 (PMC11252342; doi:10.1038/s41467-024-50389-8)
Supplement: Supplementary file 1 — Supplementary Information [file 41467_2024_50389_MOESM1_ESM.pdf]

Supplemental Information for

Epidermal growth factor-like domain 7 drives brain lymphatic endothelial cell development through integrin αvβ3

Jingying Chen[1,*], Jing Ding[2], Yongyu Li[2], Fujuan Feng[2], Yuhang Xu[2], Tao Wang[2], Jianbo He[2], Jing Cang[1] & Lingfei Luo[1,2,*]

*Correspondence to: lluo@fudan.edu.cn, jingyingchen@fudan.edu.cn

Supplemental Figures and Legends

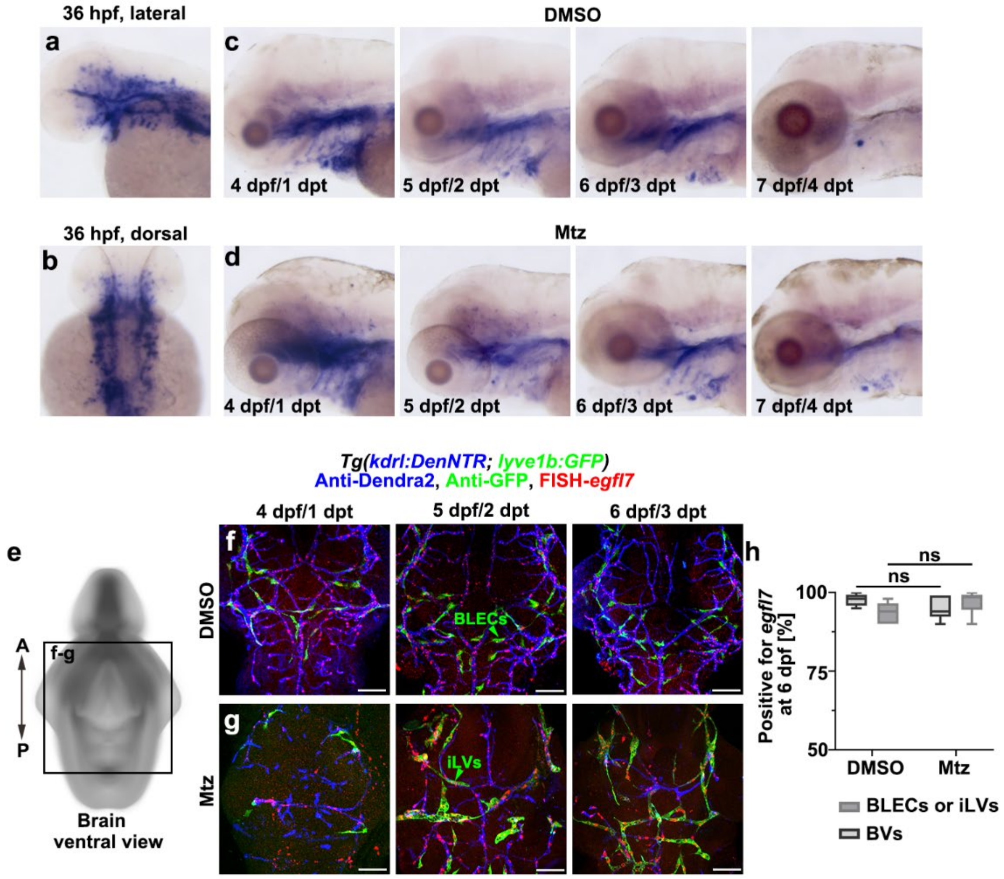

Supplementary Fig. 1 The expression pattern of *egfl7* during brain vascular regeneration. a-d Representative images of whole-mount *in situ* hybridization showing the expression pattern of *egfl7* in 36 hpf (**a, b**), after DMSO and Mtz treatment from 1 dpt to 4 dpt (**c, d**). **e** Schematic diagram showing the ventral view of larvae in f and g. **f, g** Triple labeling of FISH-*egfl7*, anti-GFP, and Dendra2 after DMSO and Mtz treatment in *Tg(kdrl:DenNTR; lyve1b:GFP)* transgenic background. Scale bar, 50 μm. **h** The statistics show the Egfl7 mRNA expressed in almost all the LECs and BECs (n=6 larvae, ns, no significance). Data are represented as boxplots with Min/Max whiskers. BLECs, brain lymphatic endothelial cells; iLV, ingrown lymphatic vessels.

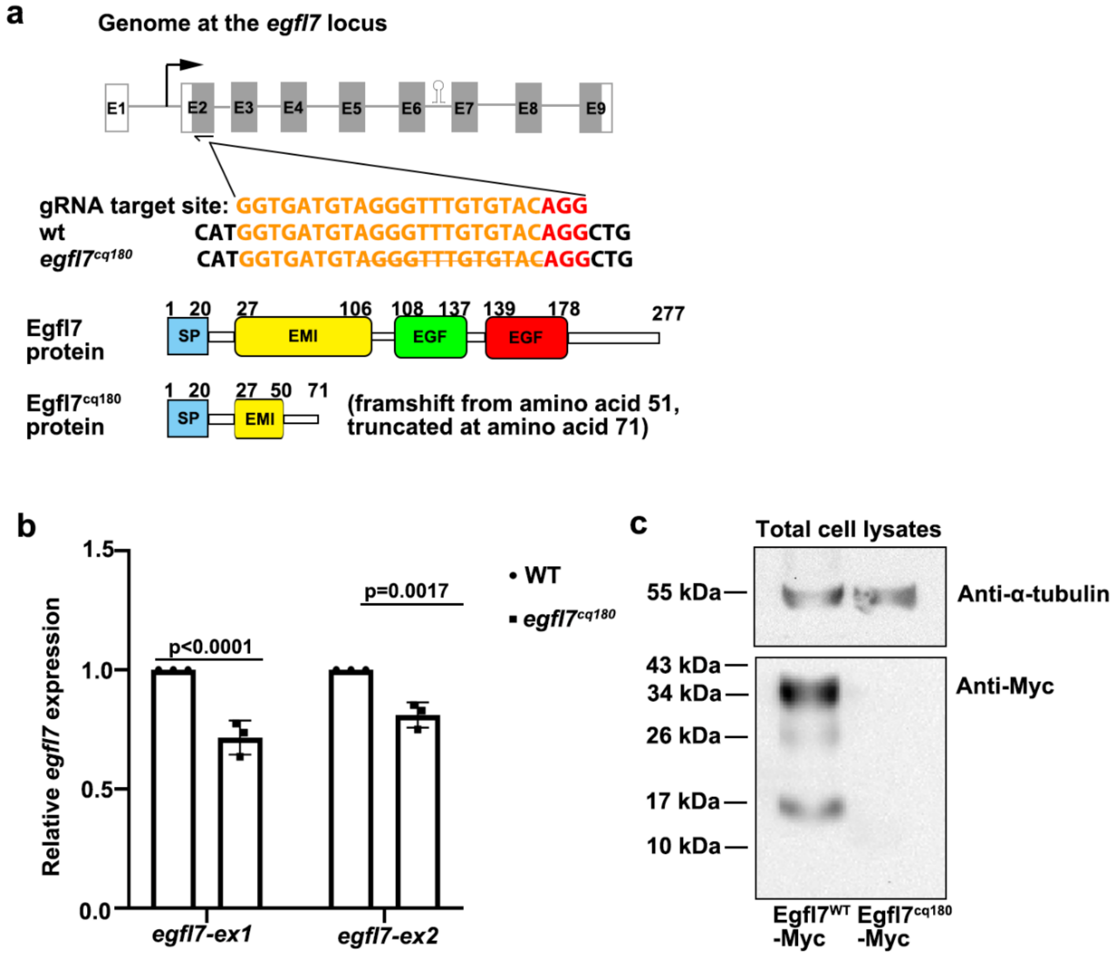

Supplementary Fig. 2 ***egfl7**^{cq180}* **mutant shows the *egfl7* mRNA degradation and reduction of protein expression. a** Schematic representation of the generation of the *egfl7*^{cq180} mutant. The orange letters, red letters, and orange horizontal lines denote the gRNA target site, the PAM site, and the deletion of 13 bp in the *egfl7* gene, respectively. Note the mutant encoded a truncated 71-amino-acid long polypeptide that contains a signal peptide and a partial EMI domain followed by a frameshift from amino acid 51. **b**, Real-time qPCR indicates the *egfl7* mRNA degradation in 5 dpf *egfl7* mutant compared to WT, expression normalized to *β-actin*. n=3 replicants, 2way ANOVA multiple comparisons test. Data are represented as mean ± SD. **c**, The *egfl7*^{cq180} mutation leads to strongly reduced protein expression. Western blot analyses of Egfl7-Myc-tag expression in transfected HEK293T cells. C-terminally tagged Egfl7-WT-myc protein (about 31 kDa) was detected in total cell lysate, whereas the truncated Egfl7-mutant-myc protein (about 8 kDa) was strongly decreased. In addition, Egfl7^{cq180} shares high similarity to the truncated protein produced in the *Egfl7*^{s981} mutant embryos in which the proteins were strongly reduced or undetectable in the

cells [29].

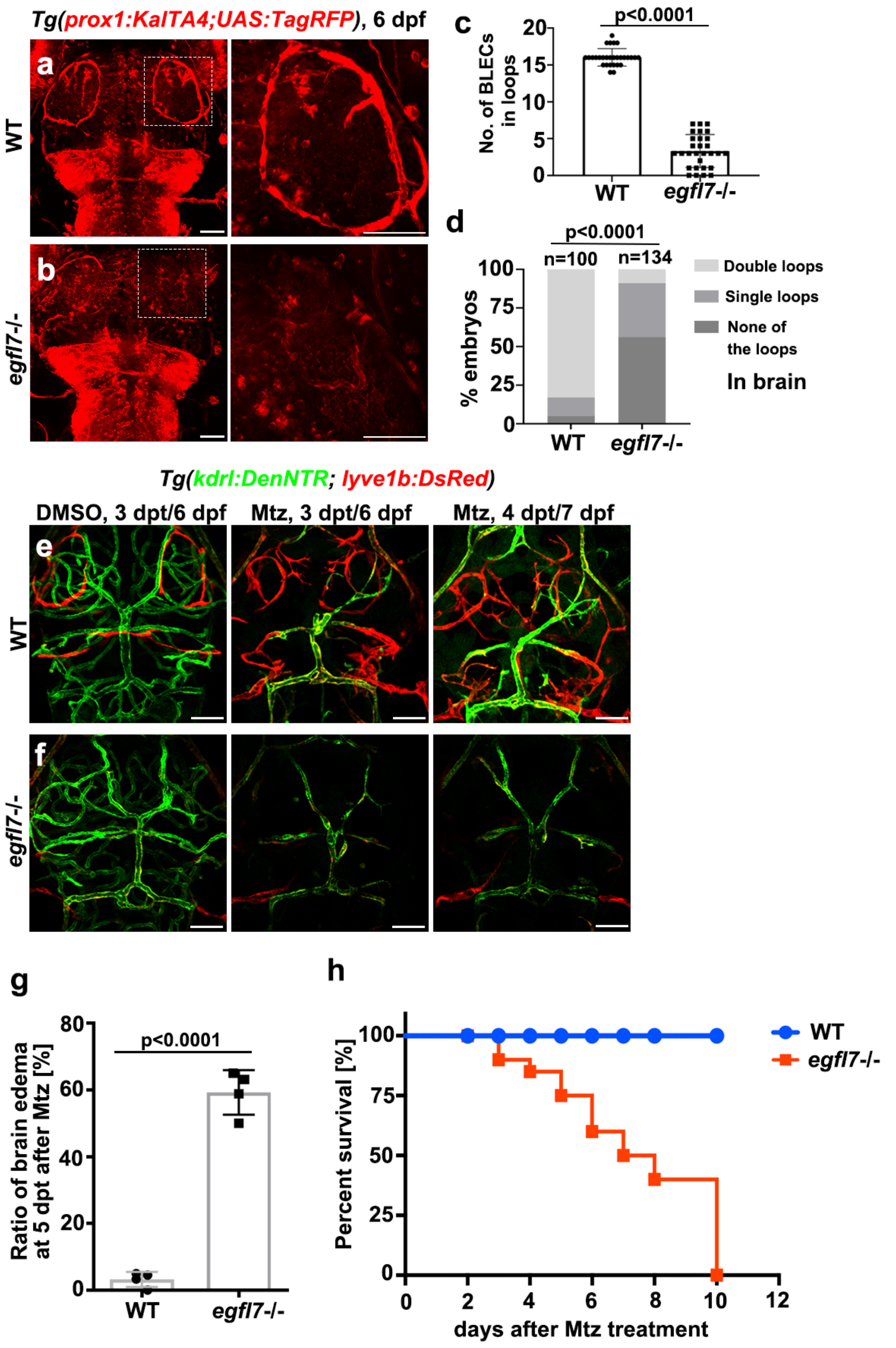

Supplementary Fig. 3 ***egfl7*** **mutation leads to brain vascular regeneration defect. a-d** Confocal images of the BLECs in *Tg(prox1:KalTA4;UAS:TagRFP)* transgenic lines at 6 dpf in WT and *egfl7* mutant, the white frames indicate the magnified BLECs loop (**a**, **b**). The statistics show the number

of BLECs in loops (**c**, n=29 embryos, two-tailed unpaired t-test. Data are represented as mean ± SD) and the percentage of embryos that have double lymphatic loops, single loops, and none of the loops in the brain (**d**, WT, n=100, *egfl7-/-*, n=134, χ^2 test). **e, f** Confocal images of brain blood vessels and BLECs under *Tg(kdrl:DenNTR;lyve1b:DsRed)* transgenic background after DMSO and Mtz treatment in WT and *egfl7* mutant. **g, h** The Statistics show the ratio of brain edema (**g**, n=4 replicants, two-tailed unpaired t-test, Data are represented as mean ± SD) and the survival rates (**h**, n=40). Scale bar, 50 μm.

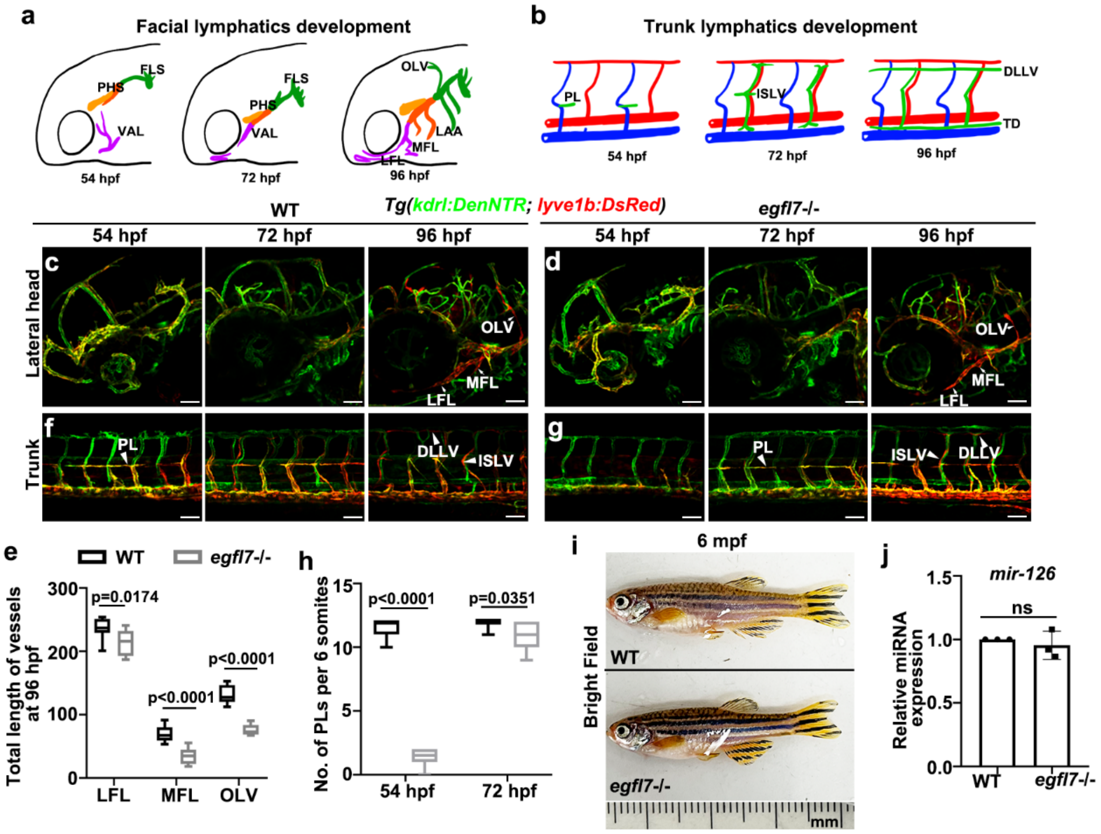

Supplementary Fig. 4 Egfl7 is dispensable for facial and trunk lymphatic development. a, b Schematic representation of facial and trunk lymphatic development from 54 hpf to 96 hpf. VA-L, ventral aorta lymphangioblast; PHS, primary head sinus; FLS, facial lymphatic sprout; LFL, lateral facial lymphatic; MFL, medial facial lymphatic; LAA, lymphatic branchial arches; OLV, otolithic lymphatic vessel; PL, parachordal lymphangioblasts; ISLV, intersegmental lymphatic vessels; DLLV, dorsal longitudinal lymphatic vessel; TD, thoracic duct. **c-f** Confocal images of the facial lymphatics, and trunk lymphatics development in *Tg(kdrl:DenNTR;lyve1b:DsRed)* transgenic lines from 54 hpf to 96 hpf in WT (**c, f**) and *egfl7* mutant (**d, g**). Scale bar, 50 μm. **e**, **h** The statistics show the total length of LFLs, MFLs, and OLVs at 96 hpf (**e**, n=8 embryos) and the number of PLs per 6 somites at 54 hpf and 72 hpf (**h**, WT, n=8 embryos; *egfl7-/-*, n=7 embryos). 2way ANOVA multiple comparisons test. Data are represented as boxplots with Min/Max whiskers. **i** Bright-field images of adult zebrafish in WT and *egfl7* mutant at 6 mpf. Note the overall morphology of adult *egfl7* mutants appears relatively normal, does not develop edema, and is viable and fertile. **j** Real-time qPCR for *mir-126* expression in WT and *egfl7* mutant (n=3 replicants, two-tailed unpaired t-test; ns, no significance). Data are represented as mean ± SD.

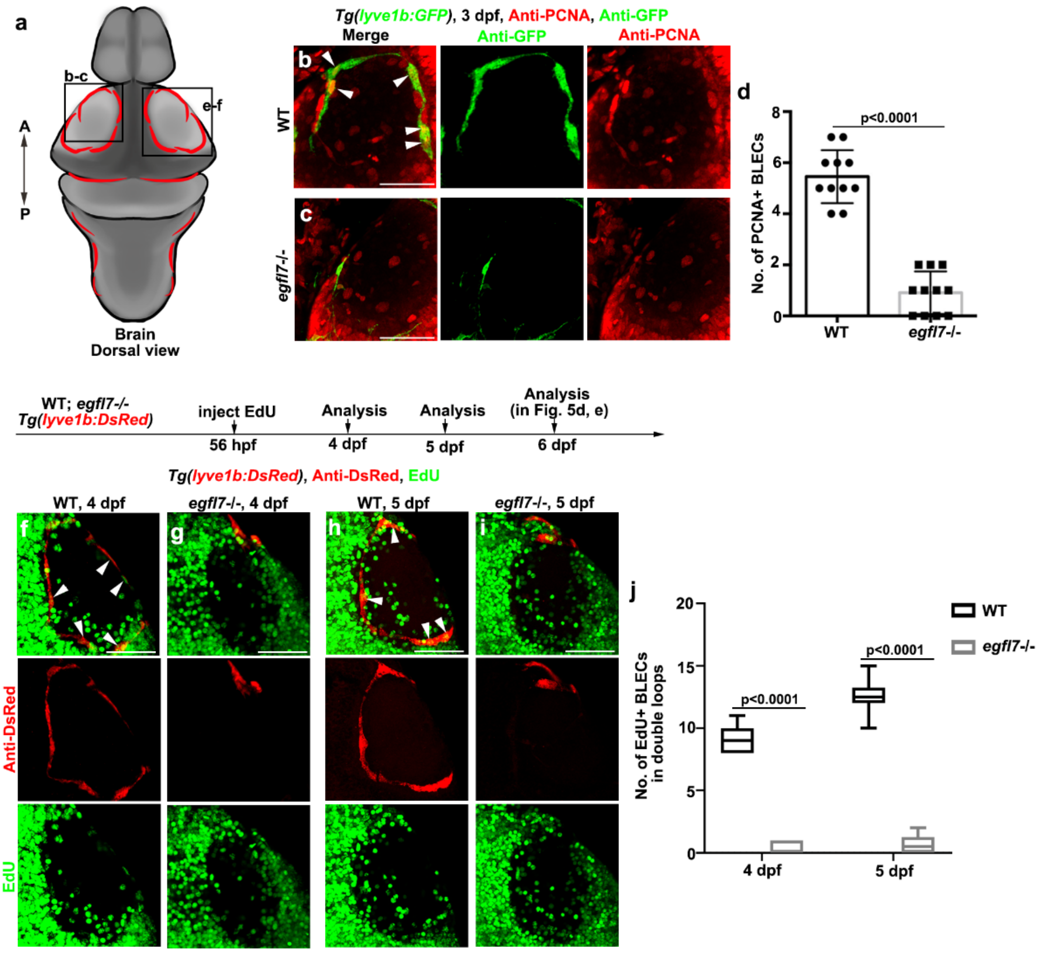

Supplementary Fig. 5 The proliferation of BLECs is reduced in the *egfl7* mutant. a Schematic diagram showing the dorsal view of larvae brain. Black frames indicate the image area of corresponding panels. **b-d** Immunofluorescence staining for BLECs and Anti-PCNA in the WT and *egfl7* mutant at 3 dpf (**b, c**). Arrowheads indicate the PCNA+ BLECs. The statistics show the number of PCNA+ BLECs (**d**, n=11 embryos; two-tailed unpaired t-test). Data are represented as mean ± SD. **e** Schematic diagram showing the experimental design for detecting proliferation of BLECs by EdU staining. **f-i** EdU staining for BLECs in WT and mutant under *Tg(lyve1b:DsRed)* transgenic line at 4 dpf and 5 dpf. Arrowheads indicate the EdU+ BLECs in WT. **j** The statistics show the number of EdU+ BLECs in double loops (**c**, n=10 brains; 2way ANOVA multiple comparisons test). Data are represented as boxplots with Min/Max whiskers. Scale bar, 50 μm.

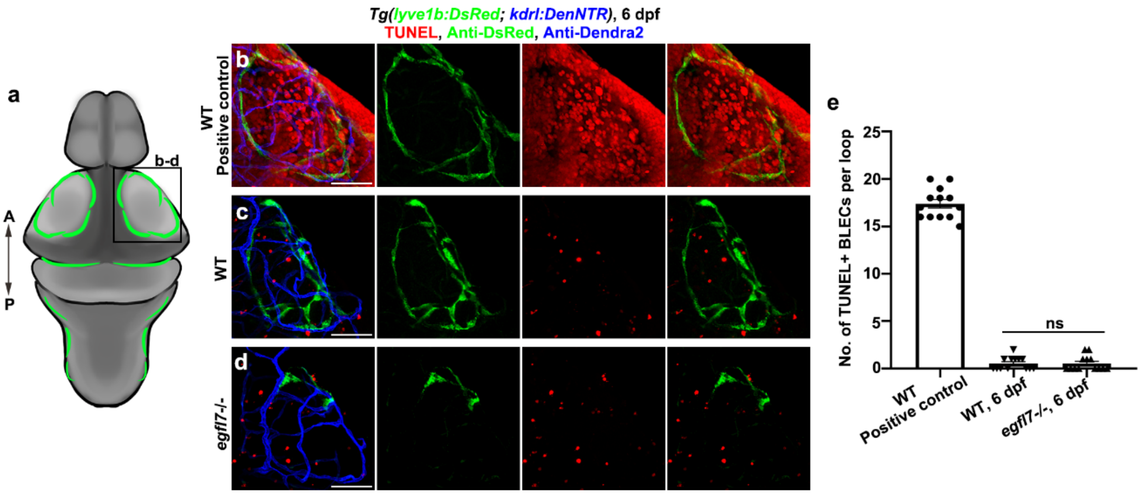

Supplementary Fig. 6 *egfl7* mutation does not induce apoptosis of BLECs. a Schematic diagram showing the dorsal view of larvae brain. Black frames indicate the image area of corresponding panels. **b** A positive control of TUNEL assay by treating a WT with DNAse to cause DNA breakage before staining. **c-d** TUNEL assay of BLECs in WT and *egfl7* mutant at 6 dpf (**c, d**). **e** The statistics show the number of TUNEL+ BLECs in the brain (n=13 embryos; two-tailed unpaired t-test; ns, no significance). Scale bar, 50 µm. Data are represented as mean ± SEM.

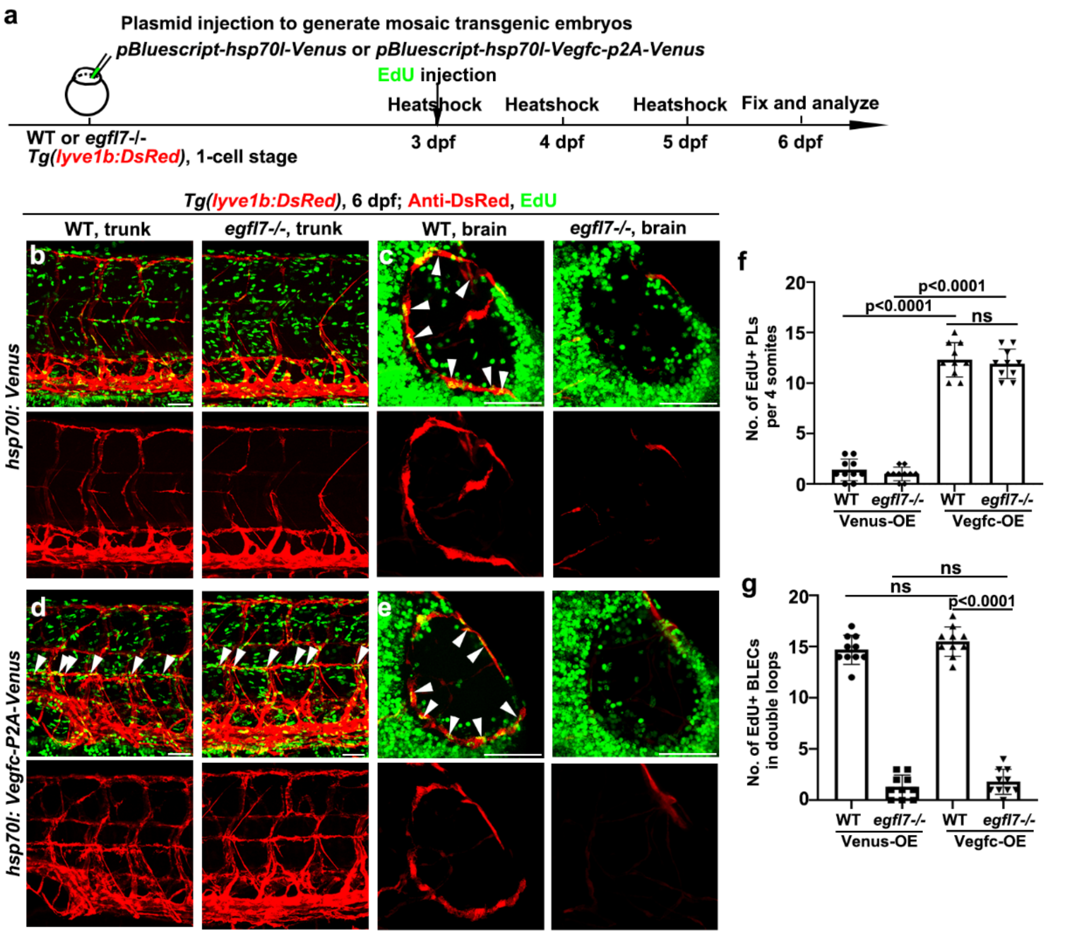

Supplementary Fig. 7 Inducing Vegfc-driven proliferation fails to rescue the absence of BELCs in the *egfl7* mutant. a Schematic diagram showing the experimental design for detecting the proliferation of LECs after the ectopic, mosaic expression of Vegfc and Venus. Illustrations of the method, transgenic lines, plasmids, and time points of heat shock, EdU injection, and analysis. **b, d** Heat shock and over-expression of Vegfc, but not Venus, causing a hyperproliferation in adjacent PLs (arrowheads) of WT and *egfl7* mutant. **f** Quantification of EdU+ PLs per four somites after ectopic expression of Vegfc and Venus in the trunk (n=10 embryos; two-tailed unpaired t-test, ns, no significance). **c, e** Compared to the heat-shock of Venus, over-expression of Vegfc in the brain does not alter the proliferation of BLECs in the WT (arrowheads) and *egfl7* mutant. **g** Quantification of EdU+ BLECs in double loops after ectopic expression of Vegfc and Venus in the brain (n=10 brains; two-tailed unpaired t-test, ns, no significance). Scale bar, 50 μm. Data are represented as mean ± SD.

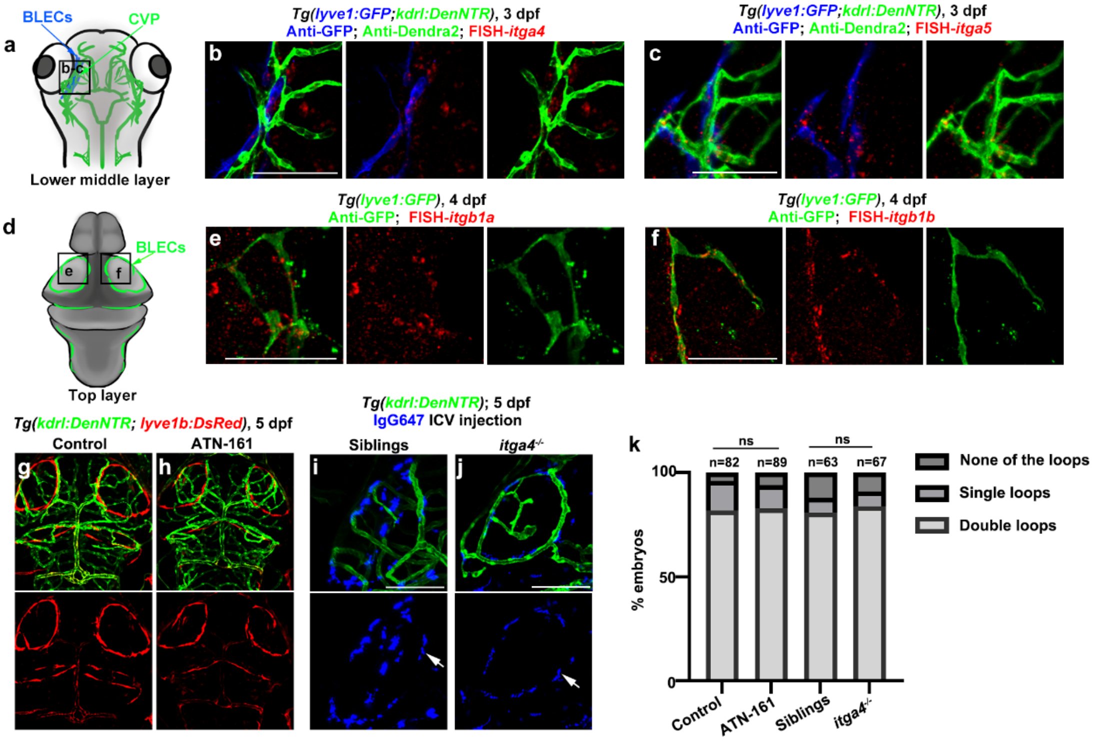

Supplementary Fig. 8 Integrin α5β1 and integrin α4β1 are dispensable for BLECs formation. **a, d** Schematic diagram showing the lower middle layer and top layer of the vessels in the dorsal view, respectively. Black frames indicate the image area of corresponding panels. **b, c** FISH and antibody staining shows *itga4* and *itga5* are expressed in BLECs in *Tg(lyve1b:GFP; kdrl:DenNTR)* transgenic background at 3 dpf. **e, f** Double labeling of FISH-*itgb1a* or FISH-*itgb1b* and anti-GFP in *Tg(lyve1b:GFP)* transgenic background at 4 dpf. Scale bar, 50 µm. **g, h** In contrast to the control group, the *lyve1b*+ BLECs showed no difference in the integrin α5β1 inhibitor-ATN-161 treated group. **i, j** Compared with the siblings, endocytosis of Alexa647-IgG was observed normally in the BLECs of *itga4* mutant after intracerebroventricular (ICV) injection at 5 dpf (arrowheads). **k** The statistics show the percentage of embryos that have double lymphatic loops, single loops, and none of the loops in the brain (Control, n=82, ATN-161, n=89, Siblings, n=63, *itga4-/-*, n=67, χ^2 test; ns, no significance). Scale bar, 50 µm.

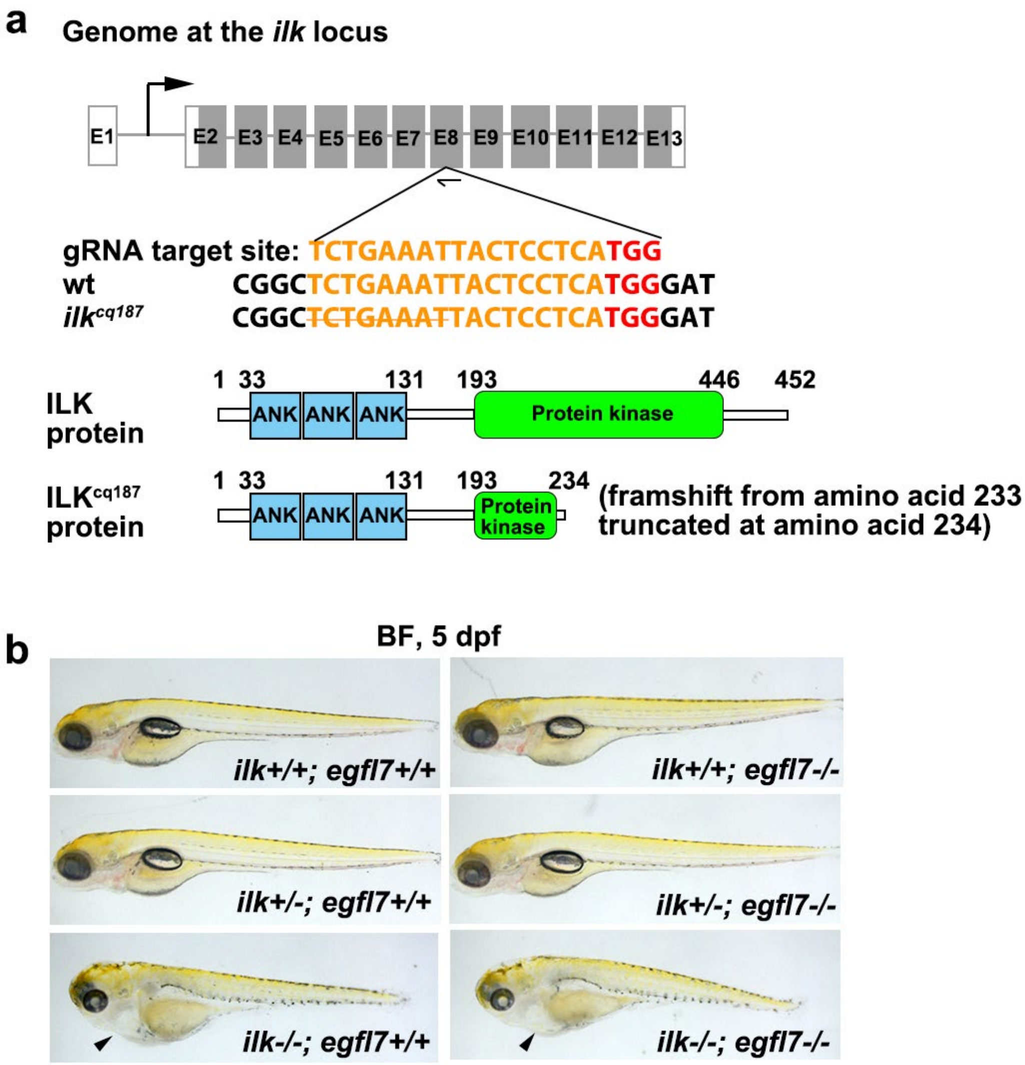

Supplementary Fig. 9 Generation of *ilk* mutant alleles. a, Schematic representation of the generation of the <u>*ilk*</u>^{cq187} mutant. The orange letters, red letters, and orange horizontal lines denote the gRNA target site, the PAM site, and the deletion of 8 bp in the *ilk* gene, respectively. Note the mutant encoded a truncated 234-amino-acid long polypeptide that contains ANK domains and a partial protein kinase followed by a frameshift from amino acid 233. **b**, Brightfield micrographs of 5 dpf larvae in lateral view. Arrows point to cardiac deformation in the presence of *ilk* mutation. Whereas the *ilk* heterozygous showed normal phenotype.

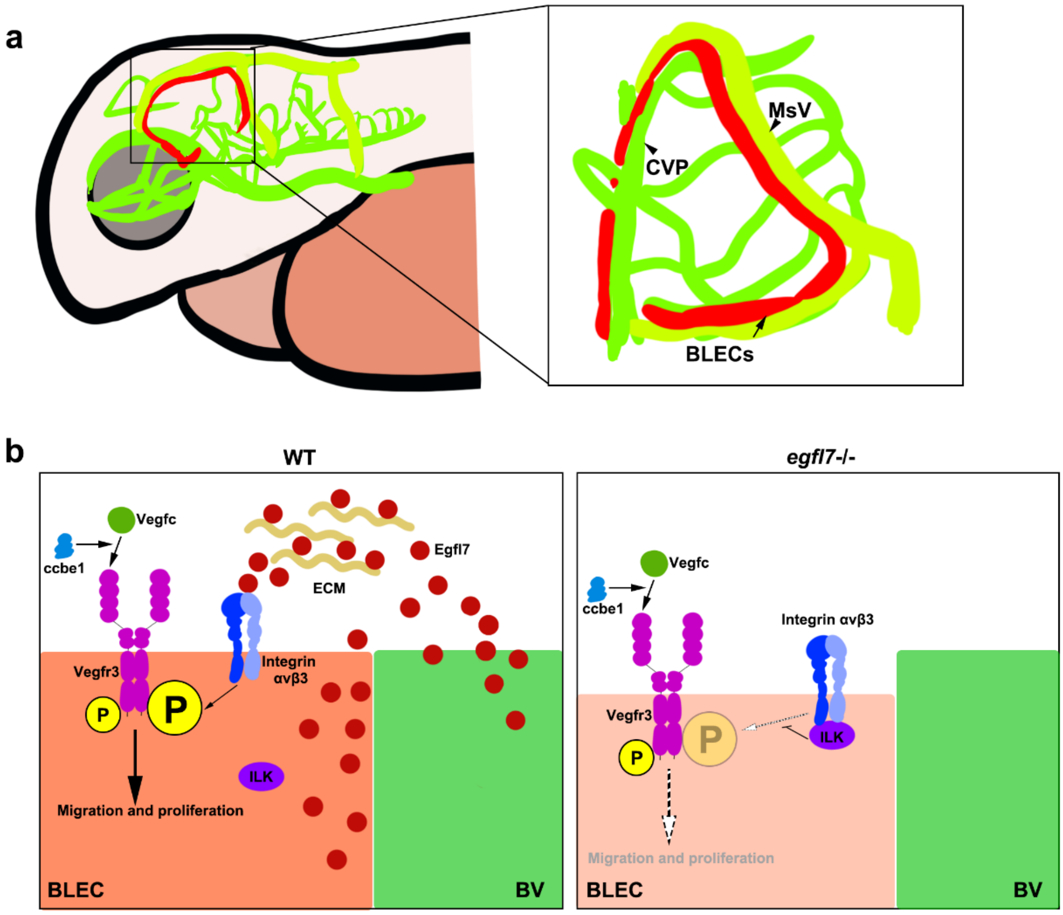

Supplementary Fig. 10 A model of Egfl7 regulates BLECs formation. a A schematic diagram shows the anatomy of the BLECs in zebrafish. Note the BLECs sprout from CVP and migrate along the MsV to form a lymphatic loop. **b** In WT, Egfl7 is deposited in the ECM upon secretion from BLECs or BVs, after BLECs attach to the Egfl7 via Integrin αvβ3, the complex of Integrin and ILK transiently disrupts, resulting in increased Vegfr3 phosphorylation ("P" in big yellow circle) and promotes BLECs migration and proliferation; In the *egfl7* mutant, Vegfr3 and Integrin αvβ3 are physically separated. ILK interacts with integrin αvβ3 to reduce Vegfr3 phosphorylation, preventing migration and proliferation of BLECs. CVP, choroidal vascular plexus; MsV, mesencephalic vein; BLECs, brain lymphatic endothelial cells; BV, blood vessel; ECM, extra cellular matrix. P, phosphorylation.
