# Supplementary material for: Epidermal growth factor-like domain 7 drives brain lymphatic endothelial cell development through integrin αvβ3
Source: Nat Commun. 2024 Jul 16;15:5986. doi: 10.1038/s41467-024-50389-8 (PMC11252342; doi:10.1038/s41467-024-50389-8)
Supplement: Supplementary file 3 — Reporting Summary [file 41467_2024_50389_MOESM3_ESM.pdf]

nature portfolio

Corresponding author(s): Jingying Chen

Last updated by author(s): Jun 24, 2024

Reporting Summary

Nature Portfolio wishes to improve the reproducibility of the work that we publish. This form provides structure for consistency and transparency in reporting. For further information on Nature Portfolio policies, see our Editorial Policies and the Editorial Policy Checklist.

Statistics

For all statistical analyses, confirm that the following items are present in the figure legend, table legend, main text, or Methods section.

n/a	Confirmed	
☐	☒	The exact sample size (*n*) for each experimental group/condition, given as a discrete number and unit of measurement
☐	☒	A statement on whether measurements were taken from distinct samples or whether the same sample was measured repeatedly
☐	☒	The statistical test(s) used AND whether they are one- or two-sided<br>*Only common tests should be described solely by name; describe more complex techniques in the Methods section.*
☒	☐	A description of all covariates tested
☒	☐	A description of any assumptions or corrections, such as tests of normality and adjustment for multiple comparisons
☐	☒	A full description of the statistical parameters including central tendency (e.g. means) or other basic estimates (e.g. regression coefficient) AND variation (e.g. standard deviation) or associated estimates of uncertainty (e.g. confidence intervals)
☐	☒	For null hypothesis testing, the test statistic (e.g. *F*, *t*, *r*) with confidence intervals, effect sizes, degrees of freedom and *P* value noted<br>*Give P values as exact values whenever suitable.*
☒	☐	For Bayesian analysis, information on the choice of priors and Markov chain Monte Carlo settings
☒	☐	For hierarchical and complex designs, identification of the appropriate level for tests and full reporting of outcomes
☒	☐	Estimates of effect sizes (e.g. Cohen's *d*, Pearson's *r*), indicating how they were calculated

Our web collection on statistics for biologists contains articles on many of the points above.

Software and code

Policy information about availability of computer code

Data collection	No software was used in the study.
Data analysis	No software was used in the study.

For manuscripts utilizing custom algorithms or software that are central to the research but not yet described in published literature, software must be made available to editors and reviewers. We strongly encourage code deposition in a community repository (e.g. GitHub). See the Nature Portfolio guidelines for submitting code & software for further information.

Data

Policy information about availability of data

All manuscripts must include a data availability statement. This statement should provide the following information, where applicable:

- Accession codes, unique identifiers, or web links for publicly available datasets
- A description of any restrictions on data availability
- For clinical datasets or third party data, please ensure that the statement adheres to our policy

This study did not generate/analyze dataset or code. All data generated and/or analysed in this study are available in the Article, the Supplementary Information, or from the corresponding author upon reasonable request. All zebrafish lines and plasmids generated in this study are available.

Research involving human participants, their data, or biological material

Policy information about studies with human participants or human data. See also policy information about sex, gender (identity/presentation), and sexual orientation and race, ethnicity and racism.

Reporting on sex and gender	*Use the terms sex (biological attribute) and gender (shaped by social and cultural circumstances) carefully in order to avoid confusing both terms. Indicate if findings apply to only one sex or gender; describe whether sex and gender were considered in study design; whether sex and/or gender was determined based on self-reporting or assigned and methods used. Provide in the source data disaggregated sex and gender data, where this information has been collected, and if consent has been obtained for sharing of individual-level data; provide overall numbers in this Reporting Summary. Please state if this information has not been collected. Report sex- and gender-based analyses where performed, justify reasons for lack of sex- and gender-based analysis.*
Reporting on race, ethnicity, or other socially relevant groupings	*Please specify the socially constructed or socially relevant categorization variable(s) used in your manuscript and explain why they were used. Please note that such variables should not be used as proxies for other socially constructed/relevant variables (for example, race or ethnicity should not be used as a proxy for socioeconomic status). Provide clear definitions of the relevant terms used, how they were provided (by the participants/respondents, the researchers, or third parties), and the method(s) used to classify people into the different categories (e.g. self-report, census or administrative data, social media data, etc.) Please provide details about how you controlled for confounding variables in your analyses.*
Population characteristics	*Describe the covariate-relevant population characteristics of the human research participants (e.g. age, genotypic information, past and current diagnosis and treatment categories). If you filled out the behavioural & social sciences study design questions and have nothing to add here, write "See above."*
Recruitment	*Describe how participants were recruited. Outline any potential self-selection bias or other biases that may be present and how these are likely to impact results.*
Ethics oversight	*Identify the organization(s) that approved the study protocol.*

Note that full information on the approval of the study protocol must also be provided in the manuscript.

Field-specific reporting

Please select the one below that is the best fit for your research. If you are not sure, read the appropriate sections before making your selection.

☒ Life sciences ☐ Behavioural & social sciences ☐ Ecological, evolutionary & environmental sciences

For a reference copy of the document with all sections, see nature.com/documents/nr-reporting-summary-flat.pdf

Life sciences study design

All studies must disclose on these points even when the disclosure is negative.

Sample size	More than ten pairs of fish were used to set one specific cross. For a cross between egfl7 homozygous parents, more than 50-150 larvae were used for analyses in each treatment. For a cross between itga4 heterozygous parents or between a heterozygote and a wild-type parents, more than 30 larvae were used for each treatment and subjected for genotyping. For a cross between ilk heterozygous and a egfl7 mutant parents, more than 100 larvae were used for each analysis and subjected for genotyping.
Data exclusions	No data were excluded from analyses.
Replication	Each experiment was repeated at least two times. The exact number of repetitions for all experiments is illustrated in the legend.
Randomization	All experiments comparing treatment groups were carried out using randomly assigned siblings.
Blinding	Phenotyping preceded genotyping in mutant analyses, hence analysis was genotype blinded. In the other experiments, the investigators were not blinded to group allocation during data collection and/or analysis.

Reporting for specific materials, systems and methods

We require information from authors about some types of materials, experimental systems and methods used in many studies. Here, indicate whether each material, system or method listed is relevant to your study. If you are not sure if a list item applies to your research, read the appropriate section before selecting a response.

Materials & experimental systems

n/a	Involved in the study
☐	☒ Antibodies
☐	☒ Eukaryotic cell lines
☒	☐ Palaeontology and archaeology
☐	☒ Animals and other organisms
☒	☐ Clinical data
☒	☐ Dual use research of concern
☒	☐ Plants

Methods

n/a	Involved in the study
☒	☐ ChIP-seq
☒	☐ Flow cytometry
☒	☐ MRI-based neuroimaging

Antibodies

Antibodies used	Primary antibodies including: GFP (1:500, Abcam#ab6658; Santa Cruz#sc9996), Dendra2 (1:500, Antibody-online#ABIN361314), Prox1 (1:1000, Abcam#ab5475), Anti-PCNA (1:500, SAB2701819), DsRed (1:500, Santa Cruz#sc101526). Secondary antibodies used in the study included donkey anti-goat IgG Alexa fluor 488-conjugated (1:1000, Invitrogen), donkey anti-goat IgG Alexa fluor 633-conjugated (1:1000, Invitrogen), donkey anti-mouse IgG Alexa fluor 568-conjugated (1:1000, Invitrogen), donkey anti-rabbit IgG Alexa fluor 647-conjugated (1:1000, Invitrogen), donkey anti-rabbit IgG Alexa fluor 488-conjugated (1:1000, Invitrogen), goat anti-rabbit IgG Alexa fluor 405-conjugated (1:1000, Invitrogen).
Validation	*Describe the validation of each primary antibody for the species and application, noting any validation statements on the manufacturer's website, relevant citations, antibody profiles in online databases, or data provided in the manuscript.*

Eukaryotic cell lines

Policy information about cell lines and Sex and Gender in Research

Cell line source(s)	cells were purchased form Cell Bank, Chinese Academy of Sciences (Shanghai, China).
Authentication	None of the cell lines used were authenticated
Mycoplasma contamination	Cell lines were not tested for mycoplasma contamination
Commonly misidentified lines (See ICLAC register)	No commonly misidentified cell lines were used in the study

Animals and other research organisms

Policy information about studies involving animals; ARRIVE guidelines recommended for reporting animal research, and Sex and Gender in Research

Laboratory animals	Zebrafish transgenic lines: The Tg(kdrl:DenNTR)cq10 20, Tg(lyve1b:GFP)cq86 21, Tg(lyve1b:DsRed)cq27 20, Tg(kdrl:CFP-NTR)cq62 20, Tg(prox1:KalTA4;UAS:TagRFP)34, Tg(egfl7:YFP) cq181, Tg(kdrl:egfl7-p2A-GFP) cq182, Tg(lyve1b:egfl7-p2A-GFP) cq183, egfl7 cq180, and itga4cas010 44 (a gift from Weijun Pan), ilkcq187 mutant were used in this study.
Wild animals	N/A
Reporting on sex	The sex of zebrafish aged from 3 dpf to 12 dpf was unknown. In adult egfl7 mutant and wild-type, both male or female fish were used for this experiment.
Field-collected samples	N/A
Ethics oversight	The zebrafish facility and study were approved by the Institutional Review Board of Southwest University (Chongqing, China). Zebrafish were maintained in accordance with the Guidelines of Experimental Animal Welfare from Ministry of Science and Technology of People's Republic of China (2006). The protocols used for animal experimentation were approved by the Institutional Animal Care and Use Committee protocols from Southwest University (IACUC, 2007). The IACUCI number is: IACUC-20231201-01.

Note that full information on the approval of the study protocol must also be provided in the manuscript.

Plants

Seed stocks	*Report on the source of all seed stocks or other plant material used. If applicable, state the seed stock centre and catalogue number. If plant specimens were collected from the field, describe the collection location, date and sampling procedures.*
Novel plant genotypes	*Describe the methods by which all novel plant genotypes were produced. This includes those generated by transgenic approaches, gene editing, chemical/radiation-based mutagenesis and hybridization. For transgenic lines, describe the transformation method, the number of independent lines analyzed and the generation upon which experiments were performed. For gene-edited lines, describe the editor used, the endogenous sequence targeted for editing, the targeting guide RNA sequence (if applicable) and how the editor was applied.*
Authentication	*Describe any authentication procedures for each seed stock used or novel genotype generated. Describe any experiments used to assess the effect of a mutation and, where applicable, how potential secondary effects (e.g. second site T-DNA insertions, mosiacism, off-target gene editing) were examined.*
