# Supplementary material for: Epidermal growth factor-like domain 7 drives brain lymphatic endothelial cell development through integrin αvβ3
Source: Nat Commun. 2024 Jul 16;15:5986. doi: 10.1038/s41467-024-50389-8 (PMC11252342; doi:10.1038/s41467-024-50389-8)
Supplement: Supplementary file 4 — Description of Additional Supplementary Files [file 41467_2024_50389_MOESM4_ESM.pdf]

Description of Additional Supplementary Files

File Name: Supplementary Movie 1

Description: Time-lapse imaging of WT and egfl7 mutant under *Tg(lyve1b:DsRed;kdrl:DenNTR)* transgenic lines from 66 to 122 hpf. The elapsed time is indicated in hours:minutes after 66 hpf. Scale bar, 50 μm.
